# An In-Depth Analysis of Medullary Strokes at a Tertiary Care Stroke Center: Incidence, Clinical and Radiological Characteristics, Etiology, Treatment, and Prognosis

**DOI:** 10.7759/cureus.43017

**Published:** 2023-08-06

**Authors:** Ahmad Muhammad, Liaquat Ali, Suhail Hussain, Abdulaziz Zafar, Ahmed Own, Syed Ghafran Ali Naqvi, Khawaja Hassan Haroon

**Affiliations:** 1 Neurology, Hamad General Hospital, Doha, QAT; 2 Neurology, Weill Cornell Medicine - Qatar, Doha, QAT; 3 Internal Medicine, Hamad General Hospital, Doha, QAT; 4 Neuroradiology, Neurosciences Institute, Doha, QAT; 5 Radiology, Hamad General Hospital, Doha, QAT

**Keywords:** modified rankin scale(mrs)., nihss (national institutes of health stroke scale), subarachnoid hemorrhage (sah), intracerebral hemorrhage (ich), mi (medullary infarcts)

## Abstract

Introduction

Medullary infarctions (MI) are a rare medical entity that is classified mainly as the more commonly lateral medullary infarcts (LMI) and the less common medial medullary infarcts (MMI). Lateral medullary syndrome, also known as Wallenberg syndrome, results when the medulla oblongata is affected and predominantly occurs secondary to atherosclerotic occlusion of the vertebrobasilar arteries. Previous studies have focused more on the anatomical, clinical, and topographical aspects of medullary infarcts. We describe the incidence of their presentation, radiological findings, etiology, treatment, and outcome at our comprehensive stroke center.

Material and method

This is a retrospective cohort study of 108 medullary stroke patients with confirmed clinical and radiological diagnoses of MI at Hamad General Hospital, Doha, between January 1, 2018 and December 31, 2020. We evaluated the electronic medical records of all stroke patients.

Result

During the selected period, a total of 2,912 ischemic strokes were reported. Of these, 843 (28.8%) were posterior circulation strokes. Only 108 (3.7%) patients had medullary strokes. Commonly encountered neurological features were dizziness (94.4%), limb ataxia (84.3%), dysarthria (44.4%), ipsilateral facial sensory loss (32.4%), headache (32.4%), contralateral limb sensory loss (25%), ipsilateral hemiparesis (24%), dysphagia (19.4%), and hiccups (13%). Most strokes reported were either minor (73% with National Institutes of Health Stroke Scale [NIHSS] 1-4) or moderate (26% with NIHSS 5-15). LMIs (87.9%) were the most common, followed by medial paramedian MI (10%). Twenty-five percent had extramedullary involvement, predominantly of the cerebellum (17.6%).

Out of the total number of patients, 44 (40.7%) had large vessel atherosclerotic disease, followed by 41 (37.6%) whose stroke was due to small vessel disease, 15 (13.8 %) due to undetermined etiology, and 6 (5.5%) due to arterial dissection. Twenty-eight patients (25.4%) underwent 48-hour Holter monitoring, which detected atrial fibrillation in two patients (1.85%). The majority of patients (98.2%, or 106 patients) received antiplatelet therapy, while 68.5% (74 patients) received single antiplatelet therapy (SAPT), and 29.6% (32 patients) received dual antiplatelet therapy (DAPT). Noteworthy is that only 2.7% (three patients) received thrombolysis as an acute reperfusion therapy. Forty-seven percent (51 patients) were discharged home (mRS 0-2), and 51.9% (56 patients) were transferred to rehabilitation facilities. Follow-up assessments were performed at the stroke clinic for 57.4% (62) of the patients. The assessments found that 46 of the follow-up patients were functionally independent at that time (mRS 0-2).

Conclusion

This is the first large local study of medullary strokes to determine their frequency, presentation, etiology, treatment, and clinical outcome. Medullary strokes represent 3.7% of total ischemic strokes at our comprehensive stroke center. MI is rare and could present with a variety of neurological and non-specific symptoms that mimic common benign conditions. Prompt and early recognition with a high index of suspicion, the use of posterior NIHSS (POST-NIHSS), and urgent MRI-diffusion-weighted imaging (DWI) of the brain in acute settings can improve early diagnosis and the rate of reperfusion therapy. Further studies are needed to enable the early recognition and treatment of medullary infarcts.

## Introduction

Stroke is the second most common cause of disability and mortality worldwide [1]. Stroke is mainly due to ischemia or hemorrhage [2]. Globally, the incidence of ischemic stroke is 68%, while that of hemorrhagic stroke is 32%. In low and middle-income countries, a higher incidence of hemorrhagic stroke has been identified [3]. Posterior circulation strokes account for 20-25% (range 17-40%) of all ischemic strokes [4]. Medullary infarctions (MI) are a rare clinical entity that can be classified into lateral medullary infarctions (LMI) and medial medullary infarctions (MMI) based on clinical and radiological patterns [5]. LMI, also known as Wallenberg's syndrome, is characterized by vertigo, diplopia, dysarthria, Horner's syndrome, and numbness (ipsilateral face and contralateral limb). LMI is commonly not associated with limb weakness. Localization in LMI is easy because of its characteristic presentation, exclusive blood supply, and a small area of involvement. Sometimes medullary infarcts can present with non-specific symptoms that mimic common benign conditions, so a high index of suspicion is needed when evaluating MI patients. Several case reports and series have described medullary syndromes. However, to our knowledge, this is the first large case series in the six Gulf Cooperation Council (GCC) countries (Qatar, KSA, UAE, Kuwait, Oman, and Bahrain) to report the incidence, clinical and radiological characteristics, etiology, treatment, and outcome of this syndrome. The research team retrospectively retrieved the electronic data of all patients admitted under our stroke service with posterior circulation strokes and reviewed the notes and imaging of all patients with medullary infarcts with clinical and radiologically established diagnoses.

## Materials and methods

This is a retrospective cohort, observational cross-sectional study conducted at Hamad General Hospital. This tertiary care hospital provides comprehensive stroke care at Hamad Medical Corporation (HMC) in Doha, Qatar. The electronic data of 108 medullary infarct patients confirmed by clinical and radiological examination between January 1, 2018, and December 31, 2020, were analyzed retrospectively. All patients with clinical and confirmed radiological (by MRI/CT head) diagnoses of posterior circulation stroke were identified, and 108 consecutive patients with medullary stroke with signs and symptoms were enrolled in the study. Three senior stroke physicians reviewed their electronic medical records and images. The physicians discussed any disagreements in clinical and/or radiological findings until a consensus was reached. Patient data were collected on age, gender, comorbidities (hypertension, diabetes, dyslipidemia, smoking, ischemic heart disease, obesity, atrial fibrillation), clinical and radiological findings, and stroke severity. The latter was defined by the NIHSS (score range from 0 to 42) as follows; minor stroke, 1-4; moderate stroke, 5-15; moderate to severe stroke, 16-20; and severe stroke, 21-42 [6]. Stroke outcome was defined by the modified Rankin scale (mRS) after treatment and rehabilitation. The mRS measures the degree of neurologic disability, where a score of 0 means no symptoms, 1 is no significant disability despite symptoms, 2 is a slight disability, 3 is a moderate disability, 4 is moderate to severe disability, 5 is a severe disability, and 6 is death [7]. Routine laboratory testing was performed according to standard clinical practice and guidelines. Testing included complete blood count (CBC), prothrombin time (PT), activated partial thromboplastin time (APTT), urea, creatinine, electrolytes, liver function test (LFT), C-reactive protein (CRP), and troponin-T. Radiological (chest X-ray, CT, CT perfusion and CTA of the head and neck, MRIs and MRAs of the head and neck) and cardiac (ECGs, echocardiograms, cardiac monitoring) screenings were also performed, among other investigations. Descriptive statistics are used to summarize and determine the sample characteristics and distribution of various considered parameters related to these patients' demographic, diagnostic, clinical, treatment, and related outcome measures. Categorical data are summarized using frequencies and percentages. All statistical analyses were done using the statistical package SPSS 24.0 (IBM Corp., Armonk, NY, USA). The study was performed according to the principles of the Declaration of Helsinki. This study was approved in January 2023 by the Medical Research Center HMC, Doha, Qatar (MRC-01-22-694). The primary objective of this descriptive research study is to determine the frequency of medullary strokes. Secondary objectives are medullary strokes' clinical features, radiological findings, treatment, and outcomes in a multicultural country.

## Results

A total of 108 medullary infarct patients (3.7%; 108 medullary stroke/2912 total ischemic stroke) with confirmed clinical and radiological diagnoses were included in this study. Their demographic information, risk factors, and clinical characteristics are shown in Table 1. The mean age was 49 years (SD 25, ranging from 24 to 74 years); 91.6% (99 patients) were male and 8.3% (9 patients) were female. Fifty-six percent (61 patients) were older than 50, while 44% (47) were younger than 50. The most common underlying risk factors were hypertension (79 patients; 73.1%), diabetes mellitus (54 patients; 50%), dyslipidemia (55 patients; 50.9%), smoking (30 patients; 27.8%), ischemic heart disease (20 patients; 18.5%), and four patients (3.7%) were obese as shown in Table 1. Notably, 7.4% (eight patients) had poor diabetic control, with HbA1c >12 mmol/l.

**Table 1:** Demographic, clinical characteristics and risk factors of medullary stroke.

Age (years) (n=108)	Frequency (n)	Percentage (%)
<50 years	47	44%
>50 years	61	56%
Gender		
Male	99	91.6%
Female	9	8.3%
Risk factors		
Hypertension	79	73.1%
Dyslipidemia	55	50.9%
Diabetes	54	50%
Smoking	30	27.8%
Ischemic heart disease	20	18.5%
Obesity	4	3.7%
Atrial fibrillation	2	1.85%
Clinical manifestations		
Symptoms		
Vertigo	102	94.4%
Headache	35	32.4%
Dysphagia	21	19.4%
Hiccups	14	13%
Diplopia	8	7.4%
Hoarseness	6	5.6%
Signs		
Limb ataxia	91	84.3%
Dysarthria	48	44.4%
Ipsilateral facial sensory loss	35	32.4%
Contralateral limbs sensory loss	27	25%
Ipsilateral hemiparesis	26	24.1%
Ipsilateral limbs sensory loss	25	23.1%
Contralateral hemiparesis	24	22.2%
Ipsilateral facial weakness	23	21.3%
Horner syndrome	15	13.9%
Contralateral facial sensory loss	14	13%
Contralateral facial weakness	12	11.1%

Neurological manifestations of medullary stroke are shown in Table 1. Symptoms at presentation in descending order of frequency were vertigo (102 patients; 94.4%), headache (35 patients; 32.4%), dysphagia (21 patients; 19.4%), hiccups (14 patients; 13%), diplopia (8 patients; 7.4%), and hoarseness of voice (6 patients; 5.6%). The most common neurological signs were limb ataxia (91 patients; 84.3%), dysarthria (48 patients; 44.4%), ipsilateral facial sensory loss (35 patients; 32.4%), contralateral limb sensory loss (27 patients; 25%), ipsilateral hemiparesis (26 patients; 24.1%), ipsilateral limb/trunk sensory loss (25 patients; 23.1%), contralateral hemiparesis (24 patients; 22.2%), and ipsilateral facial weakness (23 patients; 21.3%). Other notable signs were ipsilateral Horner's syndrome (15 patients; 13.9%), contralateral facial sensory loss (14 patients; 13%), contralateral facial weakness (12 patients; 11.1%), and positive Babinski reflex (13 patients; 12%). NIHSS severity was minor, moderate, and moderate to severe in 73.1% (79 patients), 25.9% (28 patients), and 0.9% (1 patient), respectively. Lateral medullary ischemic stroke was the most common (95 patients; 87.9%) lesion on brain imaging, while medial paramedian and bilateral paramedian medullary infarcts accounted for 10.1% (11 patients) and 1.9% (2 patients), respectively. Right and left lateral medullary infarcts were found in 42.6% (46 patients) and 45.4% (49 patients), respectively, while right paramedian and left paramedian medullary strokes were present in 8.3% (9 patients) and 1.9% (2 patients), respectively. Of the total number of patients, 75% had solely medullary ischemic strokes, but 25% had another extramedullary infarct, predominantly in the cerebellum (19 patients; 17.6%), as shown in Table 2.

**Table 2:** Etiology, NIHSS scores (on admission), infarct location, treatment, discharge, and follow-up outcome of medullary strokes. NIHSS: National Institutes of Health Stroke Scale; TPA: Tissue plasminogen activator.

Etiology of Medullary Strokes	Frequency (n)	Percentage (%)
Large vessel/vertebrobasilar atherosclerotic disease	44	40.7%
Lacunar small vessel disease	41	37.96%
Cryptogenic stroke	15	13.8%
Vertebrobasilar arterial dissection	6	5.55%
Cardioembolic stroke	2	1.85%
NIHSS score (on admission)		
Minor (NIHSS=1-4)	79	73.1%
Moderate (NIHSS=5-15)	28	25.9%
Moderate to severe (NIHSS=16-20)	1	0.9%
Medullary stroke location (MR/CT head)		
Lateral medullary	95	87.9%
Left lateral medullary	49	45.4%
Right lateral medullary	46	42.6%
Paramedian medullary	11	10.1%
Right paramedian medullary	09	8.3%
Left paramedian medullary	02	1.9%
Bilateral paramedian medullary	02	1.9%
Overall MRI /CT head infarct location		
Lateral medullary	71	65.7%
Cerebellum	19	17.6%
Medial medullary	8	7.4%
Pontine	2	1.9%
Cerebellum + pontine	2	1.9%
Bilateral paramedian medullary	2	1.9%
Occipital + cerebellum	2	1.9%
Bilateral cerebellum	1	0.9%
Spinal cord + medulla + cerebellum	1	0.9%
Treatment		
Anti-platelets therapy	106	98.2%
Single anti-platelets therapy	74	68.5%
Double anti-platelets therapy	32	29.6%
Anticoagulation	2	1.8%
TPA	3	2.7%
Discharge and follow-up		
Discharge home	51	47%
Transfer to rehab center	56	51.9%
Died	1	0.9%
Follow-up stroke clinic	62	57.4%
Lost follow up	45	41.6%

**Figure 1:**
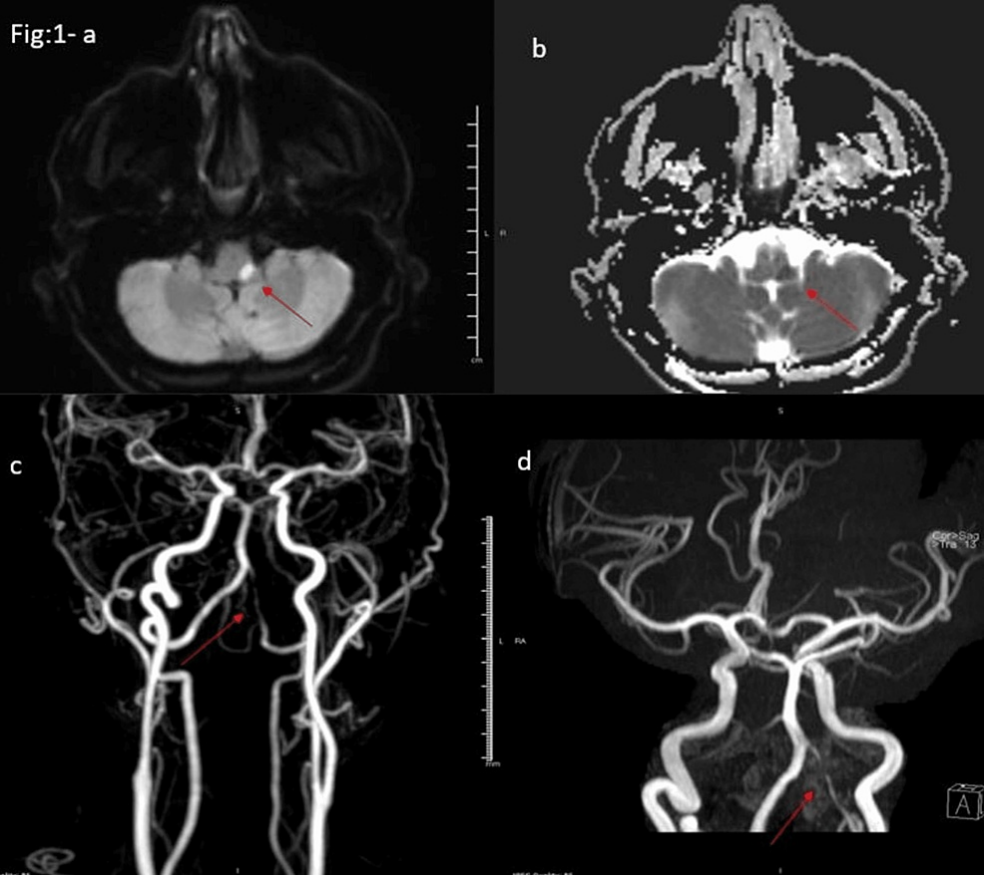
Head MRI and MRA of the head and neck vessels depicting a left lateral medullary stroke and left vertebral artery atherosclerotic disease. Head MRI images (a and b) reveal an acute left lateral medullary infarct. MRA of the head and neck (c and d) demonstrate severe atherosclerotic disease in the left vertebral artery, as indicated by the red arrow.

All 108 patients had an MRI head, MRA neck, and intracranial vessels in addition to the initial CT head, CT angiogram of the neck, and intracranial vessels, as the admitting stroke team deemed appropriate. Forty-four patients (40.7%) had large vessel atherosclerotic disease, 41 (37.6%) had infarcts due to small vessel disease, 15 (13.8%) had infarcts due to undetermined etiology as the work-up was negative, and six (5.5%) strokes were due to arterial dissection as shown in Table 2. Twenty-eight patients (25.4%) had 48-hour Holter monitoring, which detected atrial fibrillation in two patients (1.85%). Of the total number of patients, 106 (98.2%) received antiplatelet therapy. Seventy-four patients (68.5%) were given SAPT, and 32 (29.6%) were given DAPT therapy. Two patients (1.85%) were anticoagulated. Three patients (2.7%) received thrombolysis at presentation as acute reperfusion therapy, as shown in Table 2. Seven patients (6.5%) had aspiration pneumonia as a post-stroke complication. Fifty-one patients (47%) were discharged home from our stroke unit (mRS 0-2), while 56 patients (51.9%) were transferred to a rehabilitation facility, as shown in Table 2. Sixty-two patients (57.4%) attended follow-up assessments in the stroke clinic, which found that 46 patients were functionally independent (mRS 0-2) at that visit. As shown in Figure 1 (a-d), MRI and MRA of the head and neck vessels reveal an area of diffusion restriction in the left lateral medulla (seen in DWI-a and ADC map-b). Furthermore, the MRA of the neck with contrast (c) and the cranial 3-D TOF MRA (d) display severe irregular attenuation of the intracranial segment of the left vertebral artery, suggestive of atherosclerotic disease.

**Figure 2:**
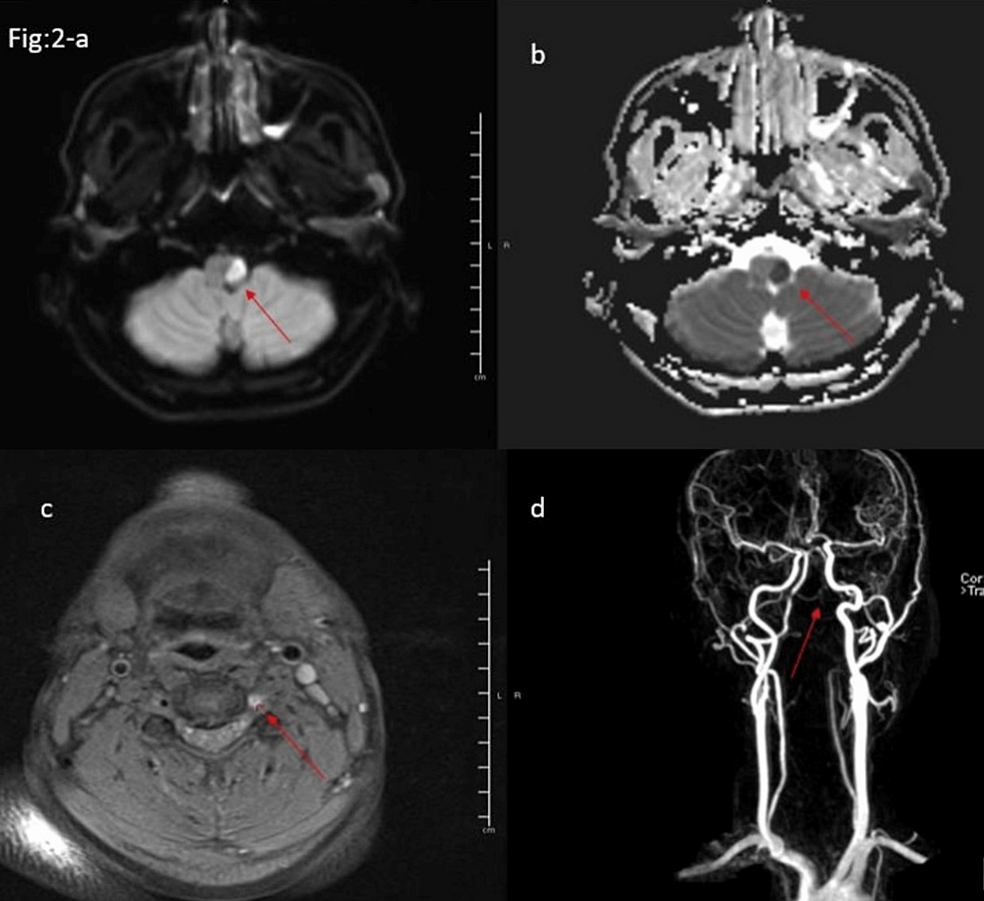
MRI of the head and MRA of the head and neck vessels, showing a left lateral medullary infarction and a dissection of the left vertebral artery. MRI of the head (a and b) shows an acute infarct in the left medulla. The MRI Axial T1 fat saturation image (c) suggests a circumferential dissection of the left vertebral artery, and MRA of the neck (d) identifies no significant flow within the left V3 and V4 segments.

As shown in Figure 2 (a-d), MRI of the head (DWI-a and ADC map-b) shows an acute left medullary infarct. Axial T1 fat saturation (c) displays a circumferential dissection of the left vertebral artery, and MRA of the neck with contrast (d) identifies no significant flow within the V3 and V4 segments of the left vertebral artery in a young male.

## Discussion

Stroke is the second most common cause of morbidity and mortality worldwide [1]. Stroke can be classified into ischemic stroke (68%) and brain hemorrhage (32%) [2, 3]. Posterior circulation ischemic strokes account for 20-25% of all ischemic strokes [4]. MI is a rare clinical entity, mainly classified as either lateral or medial MIs [5]. LMI, or Wallenberg syndrome (named after Adolf Wallenberg, 1862-1949), is the most common medullary stroke and is mainly related to vertebral artery atherosclerotic occlusive disease [8]. As there is plentiful collateral circulation, medullary infarcts represent a small proportion of all cerebral infarctions. This is the first detailed retrospective observational study of 108 consecutive patients with medullary strokes in Qatar. To our knowledge, this is the first study in the GCC countries to describe the clinical and radiological findings, etiology, treatment, and outcome of medullary stroke patients. In a study of 33 lateral medullary syndrome patients by Kim JS et al. from Asan Medical Center, Seoul, South Korea, in 1994, vertigo/dizziness (91%), gait ataxia (88%), nausea/vomiting (73%), dysphagia (61%), hoarseness (55%), Horner syndrome signs (73%), facial (85%) and hemi-body sensory changes (94%) were frequent clinical findings in LMI [9]. In our study, clinical findings in descending order of frequency were the following: vertigo/dizziness (102 patients; 94.4%), limbs ataxia (91 patients; 84.3%), limbs sensory loss, including ipsilateral (52 patients; 48.1%), facial sensory impairment, contralateral (49 patients; 45.4%), dysarthria (48 patients; 44.4%), and headache (35 patients; 32.4%). Only 21 patients (19.4%) presented with dysphagia, 15 patients (13.9%) with Horner's syndrome, and six patients (5.6%) with hoarseness of voice. In a study by Tao LS et al. in 2021, 68.7% of patients had LMI, and 28.2% had MMI. In addition, 3.1% had LMI plus MMI. This study excluded patients with extramedullary infarcts [10]. In a similar finding, our study found pure lateral medullary strokes in 65.7% of patients. However, in our study, only 7.4% of strokes were paramedian medullary strokes. This type of stroke was more common in elderly patients, as mentioned by Tao LS et al. [10]. We speculate that this lower percentage might be explained by the relatively younger age of our study's population. A study by Kim K et al. (2012), which included both medullary and extramedullary infarcts, showed 97.8% unilateral medullary and 2.1% bilateral paramedian infarcts. This finding is similar to our study, which found 98.1% and 1.9%, respectively [11]. In Kim K et al.'s study, the most common extramedullary infarcts were in the cerebellum (29.5%), while our study identified the involvement of the cerebellum was 17.7% of the patients. In Kim K et al.'s study, medullary infarcts accounted for 3.7% of all ischemic strokes, similar to ours [11]. Etiologies identified in medullary infarcts were large vessel disease (LVD), including vertebral artery dissection, a sub-type of LVD, small vessel disease (SVD), cardiac embolism (CE), and undetermined (UN) etiology. In the study mentioned above by Kim K et al., the most common etiologies were LVD (34.5%), vertebral artery dissection (9.2%), and cardioembolic (4.2%) [11]. A study by Zhang DP et al. in 2021 found LVD in 59%, SVD in 10.1%, CE in 4.5%, vertebral artery dissection in 11.2%, and unknown in 12.8% of patients [12]. A study of 130 patients with lateral medullary infarcts by Kim JS et al. in 2003 showed large vessel infarction in 50%, arterial dissection in 15%, small vessel infarction in 13%, and cardiac embolism in 5% [13].

Our study showed that 44 patients (40.7%) had large vessel atherosclerotic disease, 41 patients (37.6%) had infarcts due to small vessel disease, 15 (13.8%) had infarcts due to undermined etiology as workup was negative, and six stroke patients (5.5%) were due to arterial dissection. In our study, the most common etiologies, 78.3% (85 patients), were occlusive, atherosclerotic, and SVD, as there was a high prevalence of risk factors in our multicultural Asian population, such as hypertension (79 patients; 73.1%), diabetes mellitus (54 patients; 50%), dyslipidemia (55 patients; 50.9%), smoking (30 patients; 27.8%), ischemic heart disease (20 patients; 18.5%). Of the diabetic patients, eight (7.4%) had poor diabetes control, with HbA1c >12 mmol/l. Our study showed only six (5.5%) strokes were due to arterial dissection, compared to 9.2% in the study by Kim K et al. [11]. This low percentage is probably the result of the multiple risk factors in our population group. Of our patients, 28 (25.4%) had 48-hour Holter monitoring, which detected atrial fibrillation in two patients (1.85 %), both older than 60 years. Most of our patients (73.1%) had NIHSS severity scores indicating minor strokes, while 25.9% had scores suggesting moderate strokes. Three of our patients (2.7%) received thrombolysis. Most patients had minor symptoms, the majority presented late, and some patients had delayed diagnoses as symptoms had been attributed to other benign conditions. These factors explain why only a small percentage of patients received acute reperfusion therapy. This lack emphasizes the importance of recognizing posterior circulation strokes early, as delayed diagnosis potentially deprives an appropriate candidate of reperfusion therapy, which could leave the patient disabled. POST-NIHSS, a modified version of the NIHSS, includes an assessment of gait/truncal ataxia and bulbar signs. This version is used to improve prognostic accuracy for posterior circulation ischemic strokes with mild to moderate symptoms and a low NIHSS score (NIHSS score <10) in patients who may benefit from acute reperfusion therapies [14]. All the patients in our study underwent ECGs, with most undergoing ECGs and Holter monitoring. Most of the medullary strokes (98.2%) were treated with antiplatelet therapy. Seventy-four patients (68.5%) were given SAPT, and thirty-two patients (29.6%) received DAPT mainly due to severe atherosclerotic disease in the culprit's vessel [15]. Less than 2% were treated with anticoagulation medication, as the etiology was atrial fibrillation. Our study found that 51 patients (47%) were discharged home from the stroke unit with mRS (0-2), while 56 (51.9%) were transferred to the rehabilitation facility for an average duration of eight weeks. One patient died (0.9%) during admission due to post-stroke complications, mainly aspiration pneumonia. Only 62 patients (57.4%) attended a three-month follow-up at a stroke clinic. At this clinic assessment, 46 patients were found to be functionally independent (mRS 0-2). Forty-five patients (41.6%) did not attend follow-up at the clinic after discharge from the hospital. The likely reason is that our population consists mostly of expats. Many left for their home countries after these health events, mainly for personal reasons. In Kim JS et al.'s study of 130 pure lateral medullary strokes [13], 83% showed favorable outcomes (mRS 0-2) at a follow-up of less than three months; 95.1% of patients were functionally independent (median mRS: 1) at the last follow-up (median 17 months, IQR: 15- 18 months), while two patients died during the follow-up period. Another study by Hong Yh et al. found favorable outcomes in 89.1% and poor outcomes in 10.9% of patients [16]. In the study by Norrving B and Cronqvist S on 43 patients with LMI, 11.6% of patients died from respiratory and cardiovascular complications in the acute phase [17]. In our study, only one fatality was identified. A possible explanation for this low fatality rate could be the relatively young age of the sample and improvements in acute stroke care, specifically in the prevention of complications. Such improvements include early and prompt dysphagia screening and the adoption of appropriate measures. Posterior circulation infarction remains more challenging to recognize and treat effectively than other types of stroke [4]. This type of infarction may cause rapid neurological deterioration, worsening neurological symptoms, long-term disability, and even death, so its early recognition is crucial. Dizziness, vertigo, and imbalance are common symptoms of posterior circulation infarction but are also commonly caused by benign conditions. Therefore, detailed neurological assessment, early and prompt recognition, and appropriate acute reperfusion therapy could prevent disability, save lives, and potentially lead to a better patient outcome in medullary strokes.

Limitations

When interpreting the findings of our study, certain limitations should be considered. Firstly, the study is retrospective and could be subject to bias in clinical observation and documentation. Second, some patients had mild neurological symptoms, and a lack of knowledge of ED physicians about medullary strokes might have resulted in certain fine details being overlooked, as all data was retrieved from the electronic medical records. Finally, the outcomes available for most of our patients were at three months, which is quite a short duration when using mRS as a functional status tool. This scale is not ideal for assessing the outcomes of medullary infarcts because the mRS does not reflect symptoms such as severe sensory loss, central pain, or dizziness. However, at present, no better tool exists for the assessment of long-term outcomes in stroke patients.

## Conclusions

This is the first large local study in the GCC region on medullary strokes to determine their frequency, presentation, etiology, treatment, and clinical outcome. Medullary strokes at our comprehensive stroke center represent a frequency of 3.7% of total ischemic strokes. Medullary infarcts mimic different benign conditions, leading to missed and delayed diagnosis, and patients may not receive timely acute reperfusion therapy. Prompt and early recognition, supported by a high index of suspicion on the part of the treating medical professional, the use of POST-NIHSS, and urgent MRI-DWI of the brain in acute settings can improve early diagnosis and increase the rate of acute reperfusion therapy. Further studies are needed to enable the early recognition and treatment of medullary infarcts. 
